# Preoperative Planning Using 3D Printing Technology in Orthopedic Surgery

**DOI:** 10.1155/2021/7940242

**Published:** 2021-10-12

**Authors:** Dereje Gobena Alemayehu, Zhi Zhang, Elena Tahir, Djovensky Gateau, Dang-Feng Zhang, Xing Ma

**Affiliations:** Department of Orthopedic Surgery, The First Affiliated Hospital of Xi'an Jiaotong University, Xi'an, 710061 Shaanxi, China

## Abstract

The applications of 3D printing technology in health care, particularly orthopedics, continue to broaden as the technology becomes more advanced, accessible, and affordable worldwide. 3D printed models of computed tomography (CT) and magnetic resonance image (MRI) scans can reproduce a replica of anatomical parts that enable surgeons to get a detailed understanding of the underlying anatomy that he/she experiences intraoperatively. The 3D printed anatomic models are particularly useful for preoperative planning, simulation of complex orthopedic procedures, development of patient-specific instruments, and implants that can be used intraoperatively. This paper reviews the role of 3D printing technology in orthopedic surgery, specifically focusing on the role it plays in assisting surgeons to have a better preoperative evaluation and surgical planning.

## 1. Introduction

Three-dimensional (3D) printing is a rapidly developing technology that has gained a wide range of practical applications in health care, especially in orthopedics [1]. Although 3D printing technology has recently gained many applications in orthopedic surgery, its use is not widespread among orthopedic surgeons partially because of limited knowledge regarding the utilization of the technology. At present, 3D printing technology is not broadly available in every corner of the world.

3D printed models of computed tomography (CT) and magnetic resonance image (MRI) scans can reproduce a replica of anatomical parts that allows surgeons to preoperatively plan and rehearse complicated orthopedic procedures [2, 3]. 3D printing technology plays a crucial role in improving preoperative surgical planning in orthopedics, especially in areas that require complex 3D imagination of the underlying anatomy and determination of the size of the implant that can be used intraoperatively. The technology allows us to preoperatively create 3D structures that resemble the actual anatomy and pathology of the patients that the surgeon experiences intraoperatively. This 3D structure then enables the surgeon to plan his surgery based on the printed model to decide on the surgical approach, the method of reduction to be used, the implant size required, position and orientation of the implant, and rehearse the procedure on a 3D printed replica of the anatomical parts. These features allow the surgeon to decide on all these surgical considerations preoperatively with the aid of 3D printing technology. The application of 3D printing technology gives the advantage to reduce surgical time, limit fluoroscopy frequency, decrease the risk of infection and implant mal-positioning, decrease intraoperative blood loss, and other postoperative complications [1, 4–8]. Thus, 3D printing improves the surgeon's 3D orientation of the anatomy that is very essential for the smooth flow of the surgical procedure and improves the standard of care.

Moreover, orthopedic surgery uses implants and prosthetic materials in the treatment of musculoskeletal diseases. However, orthopedic implants and prostheses that are mass-produced at the factory level have a limited available size design to be used in all circumstances. 3D printing technology gives us the advantage of producing patient-specific implants where the implants or prostheses are 3D printed for a specific user's body thus limiting the concept of “one size fits all” for an average user [8–13]. The production of patient-specific orthopedic implants using 3D printing technology avoids the discomfort that arises from size mismatch and variations in the anatomy of certain individuals.

The applications of 3D printing technology in health care, particularly orthopedics, continue to broaden as the technology become more advanced, accessible, and affordable to every corner of the world. This review will present the role of 3D printing technology in orthopedic surgery, specifically focusing on the role it plays in assisting surgeons to have better preoperative evaluation and planning.

## 2. Preoperative Planning Using 3D Printing in Orthopedics

### 2.1. Spine Surgery

The spine has a complex anatomy, and 3D printing technology allows us to understand the complex anatomy of the spine better. Traditionally, spine pathologies are evaluated based on X-rays, CT scan, and MRI images. However, the application of 3D printing technology in the evaluation and surgical planning of spine pathology provides a better representation of the patient's anatomy and the detailed pathology to improve the standard of care given to the patients.

Preoperative planning using a 3D printed model for serious spinal deformity is the main application of 3D printing in spine surgery. 3D printing techniques can print the exact morphology of the spine for patients with spine deformity from the CT scan or MRI data. This 3D model can give the surgeon an advantage to preoperatively review the spine model and have adequate preparation, including the resources required before surgery. The surgeon can take advantage of the 3D printed spine model to preoperatively decide on the level and degree of osteotomy required, the screw trajectory, the level of fixation, and more. This feature is particularly useful for young surgeons with limited experience to shorten their learning curve. It allows them to practice the surgery on the printed spine model before going for the actual surgery. A study by Wu et al. investigated the application of the rapid prototyping (RP) technique to improve the accuracy of pedicle screw placement in patients with congenital scoliosis. They compared the accuracy and safety of pedicle screw placement using the RP technique and conventional fluoroscopy. The study demonstrated that preoperative and perioperative planning with the RP technique enhances the accuracy of pedicle screw placement. Furthermore, the study recognized the importance of using the RP technique to shorten the operative time, increasing the success of the scoliosis correction rate with lesser neurovascular complications [7].

In another study that has evaluated the accuracy of spine deformity surgery assisted with 3D printing technology, Chen et al. demonstrated that spinal deformity correction surgery has a high acceptance rate (97.1%) when assisted with 3D printing. It has superior accuracy in pedicle screw placement than free-hand and fluoroscopy-guided techniques [14]. There was no reported neurovascular complication in 173 pedicle screws implanted in 10 spinal deformity surgeries. In their study, 3D printing technology was used to create a drill template based on the bony surface anatomy and the trajectory of pedicle screws determined on CT scan images. Then, preoperative simulation surgery was conducted on 3D spine models to evaluate the safety and effectiveness of drill templates, and finally, the 3D template was applied to assist the spinal deformity correction surgery. The authors argued that 3D printing technology provides an effective alternative to expensive medical equipment, such as intraoperative navigation and robotic systems, to facilitate spinal deformity surgery.

3D printing is also used in the preoperative planning of other spine pathologic conditions such as spinal tumors, trauma, and infections. A recent study conducted by Xu et al. on patients with middle-upper thoracic spine trauma has demonstrated that preoperative planning of pedicle screw placement implemented on a 3D printed spine model improves the accuracy of pedicle screw placement, with an acceptance rate of 91% [15]. The report of this study supports the result of Wu et al. and Chen et al. regarding the advantage of 3D printing technology to improve the accuracy and reduce empirical errors in pedicle screw placement [7, 14].

Moreover, 3D printing technology has been applied in preoperative planning for the removal of spinal tumors and patient-specific implant design and production [5, 9]. Xiao and his colleagues also investigated the feasibility of spinal tumor resection based on the 3D printed model. The study asserted that preoperative planning for tumor resection based on 3D models was a successful, safe, and most effective means of managing cervical spine tumors. They pointed out that preoperative 3D printed models enable a better understanding of surgical margins to identify the relationship between the tumor and cervical spine. The application of 3D printing technologies to produce low-cost and patient-specific implants in the field of spine surgery is a promising area of innovation to achieve individualized patient care. There are reports of the use of customized prostheses designed based on patient-specific pathology and required management using 3D printing technologies. Mobbs et al. reported two cases where they used custom-designed titanium prostheses produced using 3D printing technologies. They treated two patients, one with a C-1/C-2 chordoma for whom tumor resection and vertebral reconstruction were performed using a custom-designed prosthesis, and for the other patient, a custom-designed titanium anterior fusion cage was used for the treatment of severe congenital spinal deformity. The authors reported that the use of 3D printed individualized prostheses was easy to put into the required position, facilitates surgery, shortens the operative time, and avoids further complex reconstruction.

In general, 3D printing has many applications in spine surgery that includes assistance in surgical planning, intraoperative guides with 3D printed templates, and the development of implants and prostheses customized to each patient. It offers a great potential to take us toward more personalized patient care. Besides, the 3D printing technology offers immense potential in the areas of research and education and undoubtedly will continue to evolve in the coming decades.

### 2.2. Hip and Pelvic Surgery

Surgeries involving the hip and pelvis are particularly challenging because of the complex anatomy that requires a detailed understanding of 3D anatomy from 2D images that are commonly available resources for preoperative planning. The use of 3D printing is becoming more common to assist surgery involving the hip and pelvis. 3D printing technology provides the surgeon with an opportunity to preoperatively design the surgery based on patient-specific anatomy and pathology. The surgical approach, reduction technique, fixation method, implant choice, and even the optimal size of prostheses to be placed can be decided based on simulation surgery on the 3D printed bone model. In situations where osteotomy is required, the surgeon can decide on the level and degree of osteotomy required entirely based on the 3D printed anatomical part. The use of 3D printing technology aids the surgeon in the management of malignant bone tumors where a reconstruction of anatomical parts is required after removal of the diseased part. With the help of 3D printing technology, the surgeon can preoperatively plan the surgical margin, determine the amount of diseased tissue required to be removed, and preoperatively design the ideal size and configuration of the implant required for reconstruction. This level of preoperative planning will help the surgeon intraoperatively for the smooth flow of the surgery, shorten operation time, reduce fluoroscopy frequency, and maximize patient benefit.

Fractures involving the acetabulum require restoration of the articular surface and anatomical structures to get satisfactory clinical outcomes. However, due to the complexity of the anatomy and neurovascular structures around the pelvis, surgery involving the acetabulum has always been challenging. 3D printed bone models enhance the understanding of acetabular fracture morphology and help the surgeon to determine the optimum treatment plan. Based on the 3D printed bone model, the surgeon can preoperatively determine the best surgical approach, reduction technique, and optimal implant contouring, positioning, and fixation. With the aid of 3D printing technology, surgical time can be effectively reduced in a well-planned surgery. A retrospective comparative study conducted by Hung et al. reported a 70-minute reduction in the duration of surgery when 3D printing technology was used compared to conventional planning using CT images [4]. Additionally, Hung et al. reported that the use of 3D printing technology in preoperative planning effectively reduced the complication rates and the amount of intraoperative blood loss and improved clinical outcomes postoperatively. Similarly, a recent meta-analysis that included nine case-control studies consisting of 638 patients reported that the use of 3D printed bone models for surgical planning in pelvic and acetabular fractures reduces the surgical time, blood loss, and the possibility of inadequate fracture reduction compared to conventional preoperative planning [6]. Other researchers have also described the application of 3D printing for treating fractures of the acetabulum. Preoperative planning with 3D printing has resulted in satisfactory fracture reduction and fixation and shortens the operation time by 30 minutes [16].

The application of 3D printing in the pelvis and acetabulum also extends to the management of hip and pelvic deformity. 3D printing adds valuable support in assessing the pathologic configuration of the problematic anatomic part. Wong et al. evaluated the effect of preoperative use of a 3D printed model for femoroacetabular impingement surgery on ten consecutive patients. They highlighted the relevance of 3D printed femoral and acetabular models to allow a dynamic appreciation of the site of impingement. They found that the use of 3D models in preoperative planning of femoroacetabular impingement surgery can change both the extent and location of planned osteoplasty and is more precise than planning with the conventional CT scan and MRI radiography [17]. In a study that evaluated the value of 3D printed models in understanding the classification and determining the optimal surgical approach for acetabular fractures, the authors reported that surgeons better understand, classify, and determine the optimal surgical approach in 3D printed models of acetabular fractures than X-ray/two-dimensional (2D) computed tomography (CT) and 3D reconstructions alone [18].

The use of 3D printing technology in total hip replacement has brought tremendous advantages in complex revision hip arthroplasty, postseptic arthritis, and dysplastic hip. Revision hip arthroplasty requires a comprehensive understanding of the 3D configuration of bony anatomy. 3D printing technology offers an advantage to the treating physician to have a detailed understanding of the patient's pathology (bone insufficiency, deficiency, discontinuity). It allows better evaluation and treatment with improved precision, especially for decision-making on implant selection, including determining the size and type of augment or cage requirements. In 2017, Hughes and his colleagues reported three complex revision hip arthroplasties in two patients where life-size 3D models were used to simulate the surgery, used as a template for implant selection, and determined the drill trajectory and screw positioning preoperatively [19]. The study indicated that preoperative templating using 3D model reduces surgical time, intraoperative blood loss, and improved intraoperative surgical decision-making. A separate similar study by Tserovski et al. demonstrated that implementation of preoperative evaluation based on a 3D model was important for predetermination and selection of a correct acetabular cup and augment in one patient with severe acetabular defects requiring total hip revision surgery. The authors reported preoperative planning and simulation on 3D printed models, improve surgical decision-making, and are important to obtain satisfactory clinical outcomes [20].

The applications of 3D printing technology in total hip replacement go beyond just aiding the preoperative evaluation and planning. Hao et al. reported that the advantage of three-dimensional (3D) printing technology in the reconstruction of large acetabular bone defects with 3D printed prosthesis was reported in four patients [21]. The authors reported that 3D printing hip prosthesis offers reliable reconstruction, stable fixation, and good functional recovery for revision total hip arthroplasty with a complex acetabular bone defect.

Overall, 3D printed anatomical models are particularly useful in pelvic and hip surgery for preoperative planning and surgery simulation, implant templating, and generation of 3D printed prostheses in the management of complex trauma, difficult total hip replacement, deformity correction, and beyond. The application of this technology in hip and pelvic surgery is fast growing and expected to be easily accessible and affordable as the technology continues to evolve.

### 2.3. Surgery around the Knee

3D printing technology can be applied in surgeries around the knee, such as total knee arthroplasty (TKA), osteotomy around the knee, and cruciate ligament repair. In TKA, preoperative templating on plain radiography has been traditionally used to predict the component size and prediction of postoperative limb alignment. 3D printing technology allows better prediction of the size of the femoral and tibial components. The use of 3D printed patient-specific cutting guides (PSCGs) is reported in total knee replacement (TKR) with improved accuracy of knee alignment, shorter surgical time, and decreased intraoperative blood loss. 3D printing technology enables a surgeon to predetermine the femoral valgus angle, which is one of the significant predictors of long-term survival in TKA. Traditionally, surgeons use a fixed femoral valgus angle in the proximal TKA cuts despite variation in the angle between different individuals [22, 23]. 3D printed surgical guide for patient-specific total knee replacement facilitates individualized intraoperative bone cutting for femoral and tibial components.

A randomized controlled study conducted on 80 patients by Chareancholvanich et al. indicated that the use of 3D printed PSCGs improved bone-cutting time and total operation time [10]. Similarly, the application of 3D patient-specific instrumentation (PSI) was reported to improve the surgical accuracy and safety of total knee arthroplasty in a study conducted on 52 patients by Zhou et al. [24]. As in the previous study, a separate study by Qiu et al. supported the significance of using 3D printed PSI in TKA [11]. The study compared the proximal osteotomy and distal osteotomy amount, valgus angle, external rotation angle, and tibial posterior slope angle in TKA conducted on 20 patients with the aid of 3D printed PSI and conventional instrumentation. The authors affirmed that PSI enables surgeons to quantitatively carry out a preoperative assessment and control intraoperative alignment better than the conventional instrumentation. The position of the implants in the desired position was more accurate in the PSI group, indicating the potential use of 3D printing to obtain the optimal alignment in TKA. The application of 3D printing in a knee with complex fractures and deformity to improve the clinical outcomes of knee surgery was reported in a prospective cohort study by Zhi et al. The study was conducted on 22 patients with complex knee fractures and deformities to compare the clinical outcomes, the surgical time, total blood loss, radiation exposure, and the duration of hospital stay. The authors reported that the use of 3D printing technology for surgical planning allows optimal preoperative planning and improves the surgical accuracy, safety, and total surgical time required when compared to the surgery planned with conventional radiologic images [25].

There are also reports emerging about the role of 3D printing in anterior cruciate ligament (ACL) reconstruction. Anatomic ACL reconstruction is essential to restore the function of the native ACL and obtain a desired clinical outcome. Femoral and tibial tunnels should be placed in their native anatomical location to restore knee function and stability. Anatomic ACL reconstruction requires individualized treatment based on patient-specific knee bony anatomy. Several bony landmarks have been reported by different authors to guide surgeons to accurately create a femoral and tibial tunnel in the desired position [26–31].

3D printing technology plays a major role in assisting the surgeon to preoperatively identify the anatomic bony landmarks that vary between patients and design the surgical procedure for a precise position of the tunnels intraoperatively. Ni et al. reported a method of accurate tunnel positioning for ACL reconstruction based on 3D printing technology on 20 cadaveric knees [32]. They used CT scan data to establish a 3D knee model, which was then used to design a resin template to guide femoral and tibial tunnel placement at ACL footprint. They observed that the deviation between the planned and actual drilled tunnel positions was less than 0.6 mm with high accuracy. The finding supports the use of 3D printing technology to improve the accuracy of tibial and femoral tunnel placement in ACL reconstruction. Recently, Liu and his colleagues did a randomized controlled trial to investigate the use of a 3D printed individualized navigation template in anatomic ACL reconstruction [33]. The study compared the accuracy of ACL reconstruction between a conventional operation and operation assisted by a 3D printed individualized navigation template in 43 patients. They reported that the tunnel position in the 3D group was accurate. In contrast, there was a deviation in the conventional group where the femoral tunnel was positioned more inferior and shallower, and the tibial tunnel was positioned closer to the anterior and medial edge of the tibial platform. Besides, they reported the advantage of using the 3D template to save intraoperative tunnel positioning time. However, there was no difference in knee scores between the two groups.

3D printing technology also has practical applications in osteotomy around the knee. Medial opening wedge high tibial osteotomy (OWHTO) is a common osteotomy procedure performed for medial compartment osteoarthritis [34]. Osteotomy around the knee limits damage to the knee joint by redistributing stress from the overloaded compartment to the relatively healthy side. This load redistribution is accomplished by realigning the weight-bearing axis to unload the overloaded compartment. Preoperative planning for high tibial osteotomy requires careful evaluation of the mechanical and anatomical axes of the lower extremities to get the desired postoperative alignment. Conventional preoperative planning in OWHTO involves the determination of the amount of correction required on a full weight-bearing leg radiograph using a picture archiving and communication system (PACS) [35, 36].

Despite the improvement in surgical accuracy in OWHTO, there are considerable reports of a tendency toward undercorrection, and there are ongoing efforts to improve the surgical accuracy in modern OWHTO [37–41]. Alemayehu et al. reported that the amount of compression required to fix the plate at the osteotomy site depends on the severity of the proximal tibial varus deformity. However, this could also result in undesired correction loss due to overcompression that is required in such patients [41]. The application of 3D printing technology to improve preoperative planning and surgical accuracy in HTO was investigated by some authors. 3D printing allows multiplanar morphological assessment of the proximal tibia better than the conventional techniques. There are also reports on the role of 3D planned PSI in OWHTO.

In an investigation that compared the accuracy of the preoperative planning method using a 3D printed model with PACS in 40 patients who underwent HTO, Kim et al. indicated that the use of a 3D printed model gives a more accurate correction for successful results [42]. Van Genechten et al. used a 3D printing technique to perform 10 OWHTO using customized 3D printed wedges that were designed based on the intended osteotomy opening [8]. 1-year follow-up results of their study indicated that 3D printing can be safely applied in OWHTO with excellent accuracy outcomes and lesser complications. The use of 3D printed PSI was described as a helpful tool in achieving successful outcomes. Fucentese et al. evaluated the surgical accuracy of OWHTO using 3D printed PSI, and the results of his study showed accurate correction of the mechanical leg axis in the 3D group [12]. Similarly, a recent study by Jeong et al. explored the use of 3D printed plating for patient-specific HTO in a patient with prior anterior cruciate ligament reconstruction [43]. They used a patient-specific surgical guide and plate to perform HTO. They indicated that 3D printed PSI can be used successfully to obtain accurate and efficient correction of varus malalignment while accommodating preexisting implants. The 6-week clinical outcome was reported to be satisfactory.

All in all, 3D printing technology has a wide range of practical applications for surgery around the knee. It is increasingly utilized to assist surgeons to have a better preoperative plan, produce surgical guides, and patient-specific instrumentation that could improve the standard of care given to patients.

### 2.4. Foot and Ankle Surgery

Foot and ankle surgeons often deal with complex foot and ankle injuries and deformities. The information that can be obtained from 3D printed models is instrumental for the evaluation of foot and ankle pathology and assists the surgeon in preoperative planning and decision-making on management. Several studies have shown that 3D printed models can be effectively employed in complex foot and ankle surgery.

A study by Jastifer and Gustafson reported a case of malunion of the fibula with posterior translation relative to the talus treated with the aid of 3D printing technology. They mentioned the use of this technology for preoperative planning and surgical simulation for correction of the foot and ankle deformity [44]. Ozturk et al. presented the clinical outcome of 10 patients with hallux valgus deformity that were treated with the help of 3D printing technology to design the hallux valgus osteotomy [45]. They recognized the importance of 3D models to improve the surgeon's perception of 3D information. Patient-specific osteotomy can be designed with improved postoperative foot function using this technology. Similarly, Xu et al. carried out a clinical study to explore the effectiveness and advantage of using 3D printed navigation templates in moderate and severe hallux valgus osteotomy [46]. Their results indicated that the 3D template-assisted group when compared with traditional Ludloff osteotomy has a higher American Orthopedic Foot and Ankle Society Score (AOFAS) and a lesser rate of first metatarsal length shortening. They supported the use of a 3D printed navigation template that assisted hallux valgus osteotomy to provide accurate preoperative planning and effective surgery.

Foo and Kwek assessed the role of life-sized 3D models to assist surgeons in visualizing CT images, aiding preoperative planning of tibial plafond fractures [47]. The study was focused on whether surgeons consider the 3D models useful, easy to use, and more accurate than conventional planning. Six surgeons have participated in the study and commented on the usefulness of simple and complex tibial plafond fracture models. The study concluded that 3D printed models are easy to use in preoperative planning of tibial plafond fractures with better accuracy. The advantage of using 3D printing technology in the treatment of calcaneal fractures was reported in a comparative study between conventional and 3D-assisted surgery [48]. Zheng et al., in their study on 75 patients, reported a significant advantage of 3D printing to shorten the duration of surgical operation, decrease total intraoperative blood loss, and the frequency of intraoperative fluoroscopy exposure. Moreover, a better radiographic result was observed in the 3D printing group postoperatively and at the final follow-up. Ozturk et al., in their comparative study between conventional and 3D-assisted calcaneal surgery, reported the benefit of using 3D printed models to allow a better preoperative plan and improve the outcome of treatment in displaced intra-articular calcaneal fractures in 37 patients involved in the study [49].

3D printing technology was also reported to assist in surgical debridement of symptomatic bone cysts of the ankle and foot. A comparative study on 21 patients by Zhang et al. where arthroscopic debridement of bone cysts was conducted with the aid of 3D printing technology in 11 patients and fluoroscopy in 10 patients reported that the use of 3D printing technology and template guide reduces the time taken to establish the arthroscopic approaches and the times of intraoperative fluoroscopy as well as intraoperative bleeding. However, there were no significant differences observed in the clinical outcome measures of the Visual Analog Scale and AOFAS Scores at the final follow-up [50].

Chung et al. and Yao et al. reported the use of 3D printing technology in minimally invasive surgery (MIS) of calcaneal fractures [51, 52]. Chung et al. described the use of a real-sized 3D printed calcaneal model as a preoperative and intraoperative tool for MIS of calcaneal fractures. They stressed the advantage of preshaping the calcaneal plate based on the real-size 3D printed model for adequate MIS calcaneal fixation. Furthermore, they suggested the use of a 3D printed model of the healthy calcaneus to evaluate the reduction by comparing it with the fractured calcaneus. Similarly, Yao et al. introduced the use of MIS for calcaneal fractures via the sinus tarsi approach with the aid of a 3D printing technique. The 3D reconstructed model of the bilateral calcanei was used to simulate the placement of the screw, using the healthy calcaneus as a control. The MIS plate was preshaped based on the 3P printed model to fit the lateral wall of the calcaneus. The result of 25 patients who underwent this procedure has shown that the MIS technique based on 3D printing improved the accuracy of screw placement, the reduction rate of the posterior articular surface, and the overall shape of the reduced calcaneus and increased the precision of the MIS for calcaneal fractures treated via the sinus tarsi approach.

Kadakia et al. presented clinical applications of custom 3D printed implants in complex lower extremity reconstruction in a series of cases [53]. They demonstrated that 3D printed cages can serve as an augment for tibiotalocalcaneal (TTC) arthrodesis in failed total ankle arthroplasty. The 3D printed cage was used to provide structural support and match the anatomy of the patient with a collapsed native talus with a large bone defect. They also presented a case of total talus arthroplasty in the setting of talar avascular necrosis. A 3D printed custom implant, which was designed based on CT scan images of the contralateral talus, was used to undergo a total talus arthroplasty in a 45-year-old female patient. Similarly, they reported the use of a 3D printed titanium cage in a patient with navicular bone loss from ballistic injury to undertake medial column arthrodesis. Similarly, Duan et al. have investigated the feasibility of 3D printed customized templates in assisting subtalar joint arthrodesis [54]. The authors reported that the use of 3D printed template guides reduces the time required to drill and position the Kirschner wires in the correct place. The operation time, as well as intraoperative radiation, was reduced when compared to the conventional group where fluoroscopy was used to assist the surgical procedure.

To sum up, 3D printing technology brings solutions to complex and challenging foot and ankle surgery. It allows adequate preoperative planning, surgical simulation, and design of customized implants that conform to anatomy, and patient-specific instrumentation for the precise correction of deformity and management of challenging foot and ankle problems.

### 2.5. Surgeries Involving the Upper Extremities

Similar to the applications of 3D printing technology to other subspecialties in orthopedics, the information that is obtained from 3D printed models can be used to improve the precision and clinical outcome of surgical procedures involving the upper limbs.

3D printing allows better evaluation of glenoid morphology than conventionally employed CT scans for total shoulder arthroplasty (TSA). Al Najjar et al. examined the role of 3D scapular models for assessing glenoid morphology. In their study that involved 32 patients scheduled for TSA, they noted that the technology is a valuable and practical tool for assessing glenoid morphology and orientation. The 3D printed scapular models were an accurate reflection of scapular anatomy that was vital for preoperative planning for TSA [55].

Preoperative planning with 3D printing technology facilitates the management of complex fractures involving the proximal and distal humerus [56, 57]. Studies comparing conventional and 3D printing-assisted surgeries of complex proximal and distal humeral fractures have indicated that the use of a 3D bone model allows a better visual display of the direction and severity of fracture-dislocation. The study supported the use of 3D printing technology to facilitate preoperative diagnosis, surgical planning, and preselection of desired implants.

The application of patient-specific 3D printed surgical guides in the correction of cubital varus deformity has been investigated by several authors. Takeyasu et al. reported on a series of 30 patients for whom custom-made 3D printed surgical guides were used for correction of cubitus varus deformity with. They reported excellent clinical outcomes in 90% of the patients [58]. Similarly, a comparative study conducted on a series of 34 patients to evaluate the accuracy and effectiveness of cubitus varus deformity correction surgery with individualized 3D printed navigation templates has indicated that the use of 3D printed templates simplifies the procedure, reduces operation time, and improves accuracy [59]. Besides, the use of augmented reality and 3D printing in preoperative planning in total elbow arthroplasty (TEA) was reported to result in greater accuracy of prosthetic placement than convention preoperative planning [60].

Moreover, 3D printing has useful applications in the management of wrist problems. The complex anatomy of the wrist joint makes surgical treatment involving the wrist challenging. Surgical treatment of complex scaphoid fractures requires adequate reduction and proper implant positioning to achieve satisfactory clinical outcomes. However, achieving stable fixation is technically challenging because of the complex geometry and position of the scaphoid and poor blood supply [61]. The use of 3D printed models and surgical guides allows surgeons to have detailed preoperative planning and can guide surgeons to accurately place scaphoid screws. Jew et al. presented a series of complex cases of scaphoid fractures that were treated with the help of 3D printing. They have shown the importance of preoperative planning with 3D printed models to gain a better understanding of 3D size, shape, and fragment geometry in complex scaphoid fractures [62]. Moreover, Guo et al. in a cadaveric study that evaluated the accuracy of an individualized 3D printed guiding template for scaphoid fracture fixation found that the technique assists for accurate placement of scaphoid screws [63].

## 3. The Future of 3D Printing in Orthopedics

It is recognized that the application of 3D printing in orthopedics is promising and is expected to advance the standard of care in orthopedic patients. We encourage surgeons to consider the use of 3D printing technology for complex cases in orthopedic surgery that requires adequate preoperative planning and a detailed understanding of 3D anatomy.

Nevertheless, the application of 3D printing technology in health care is not without challenges. The process of 3D printing takes a lot of time depending on the size of the anatomic model that is printed and the printing technique that is being used. Completion of a life-size 3D model of the hip and pelvis usually requires days which makes it challenging the application in emergency trauma cases. Therefore, the usage is limited to elective surgeries. 3D printing uses computer-aided design (CAD) packages that are used to make a 3D model. To apply 3D printing in health care, the health worker needs to be familiar with the software programs that are used to make 3D models. However, the use of computer software programs requires advanced skills and training. Therefore, considering the importance of 3D printing technology to health care, particularly orthopedic surgery, training is required to familiarize surgical residents and surgeons with the software and basic principles of 3D printing technology. Besides, working closely with the team from the radiologic workstation helps the clinicians to solve problems related to skills required for generating the 3D anatomic structures and creating printable files as well as the printing process.

Another challenge pertaining to the application of this technology in orthopedics is the requirement of high-quality materials for 3D printing in health care, especially implants and prostheses. The materials used are not available for wide use at the hospital level. Moreover, there should be a mechanism to evaluate the standard of material used for implant and prosthesis printing using the technology.

## Data Availability

No data are required.
